# Pelvic Plexus Block Versus Periprostatic Nerve Block for Ultrasound-Guided Prostate Biopsy: A Meta-Analysis

**DOI:** 10.3389/fonc.2021.655906

**Published:** 2021-05-13

**Authors:** Hui Ding, Zhongyun Ning, Hongwu Ma

**Affiliations:** ^1^ Department of Urology, Key Laboratory of Diseases of Urological System Gansu Province, Gansu Nephro-Urological Clinical Center, Lanzhou University Second Hospital, Lanzhou, China; ^2^ Department of Emergency, Lanzhou University Second Hospital, Lanzhou, China

**Keywords:** pelvic plexus block, periprostatic nerve block, prostate biopsy, prostate cancer, meta-analysis

## Abstract

**Background:**

To relieve prostate biopsy-related pain, various local anesthetic methods have been used. The best approach was periprostatic nerve block (PNB) in the past decade. Recently, pelvic plexus block (PPB) was employed to ultrasound-guided prostate biopsy. Compared with the PNB, the PPB may block a more extensive area. Therefore, PPB may be more effective in relieving prostate biopsy-related pain. However, several prospective randomized controlled trials (RCTs) comparing PPB and PNB drew conflicting conclusions, so we compared the difference of pain control between PPB and PNB for prostate biopsy.

**Methods:**

The following databases were retrieved up to October 2020: PubMed, Chinese biomedicine literature database, the Cochrane Library, China National Knowledge Internet databases, Wan fang databases and Google Scholar. Only the RCTs were included. The main outcome measures were Visual Analog Scale (VAS) score and complications. The literature quality and extracted data were evaluated by two authors independently. The software Review Manager (version 5.3) was used to perform the data analysis that comparing the difference of VAS score and complications between PPB and PNB.

**Results:**

After screening, six articles including 336 patients from PPB group and 337 patients from PNB group were performed meta-analysis in this study. The results showed that there were no significant difference of pain control in probe insertion and local anesthetic injection between PPB and PNB, while compared with PNB, patients with PPB experienced less pain during biopsy and 30 min after biopsy, respectively(MD = −0.57, 95% CI: −1.11 to −0.03, Z = 2.06, P = 0.04; MD = −0.21, 95% CI: −0.40 to −0.02, Z = 2.15, P = 0.03). In subgroup analysis, the pooled results showed that PPB was superior to PNB in 12-cores biopsy (pooled MD = −1.16, 95% CI: −1.61 to −0.71, P < 0.00001), and more than 40-ml prostate size, regardless of transrectal or transperineal prostate biopsy. The reported major complications were urinary retention, hematuria, infection and hemospermia. The pooled results showed that there were no obvious difference in complications between PPB group and PNB group.

**Conclusions:**

Overall, this meta-analysis suggests that PPB provides safe and effective pain control of ultrasound-guided prostate biopsy, and PPB is superior to PNB. In future, it also needs more high quality, large samples RCTs to verify.

## Introduction

Prostate cancer is the most common malignancy in men worldwide, with approximately 4% incidence in the males’ lifetime (1). In the United States, the diagnosis rate is one in seven men (2). Currently, prostate biopsy is the gold standard for clinical diagnosis of prostate cancer. Transrectal ultrasound (TRUS)-guided 12-core biopsy is the most common biopsy protocol (3). However, multiple biopsy cores lead to a higher incidence of infection, hematuria, and pain (4–7).

The affecting factors of the pain are mainly included the size of TRUS probe and the number of biopsies in prostate cancer patients (8). To relieve biopsy-related pain, various local anesthetic methods have been used. The previous meta-analyses confirmed that the best approach was periprostatic nerve block (PNB), which injecting lidocaine into the region of bilateral junctions between the bladder, prostate, and seminal vesicle (9, 10). Recently, a previously local anesthetic method, pelvic plexus block (PPB) was employed to ultrasound-guided prostate biopsy through blocking the area lateral to the tip of the seminal vesicles (11). Compared to the periprostatic area, the PPB may block a more extensive area. Therefore, PPB may be more effective in relieving prostate biopsy-related pain (12). However, previous meta-analyses (13, 14) only have included two studies and the number of cases is relatively small, and only VAS scores were compared, no complications were compared. In the past few years, more prospective randomized controlled trials (RCTs) comparing PPB and PNB drew conflicting conclusions (12, 15–19). Thus, in order to get more reliable evidence to guide clinical practice, a meta-analysis is necessary to determine whether PPB is superior to PNB for pain control during ultrasound-guided prostate biopsy.

## Methods

### Search Strategy

The following databases were retrieved: Pubmed, Chinese biomedicine literature database, China National Knowledge Internet databases, Wanfang databases, Google Scholar and the Cochrane Central Register of Controlled Trials via the Cochrane Library on October, 2020. Search terms combined patient-related terms (prostate cancer biopsy) and intervention terms (pelvic plexus block or PPB and periprostatic nerve block or PNB).

### Inclusion Criteria and Study Eligibility

We evaluated the records in accordance with the Preferred Reporting Items for Systematic Reviews and Meta-Analyses statement. We defined study eligibility using the PICO (patient population, intervention, comparator, outcomes), and setting methods. Included studies were those comparing pain control between PPB and PNB in patients undergoing ultrasound-guided prostate biopsy. Men with a history of previous biopsies, chronic prostatitis, chronic pelvic pain, inflammatory bowel disease, anorectal problems, active urinary tract infection, bleeding disorder, neurological conditions and local anesthetic allergy were excluded. The search was performed with written in English or Chinese. Only the RCTs were included. When two or more studies were reported by the same institution and/or authors in overlapping time periods, the most recently published report that included the largest number of patients was used.

### Data Extraction

**Figure 1:**
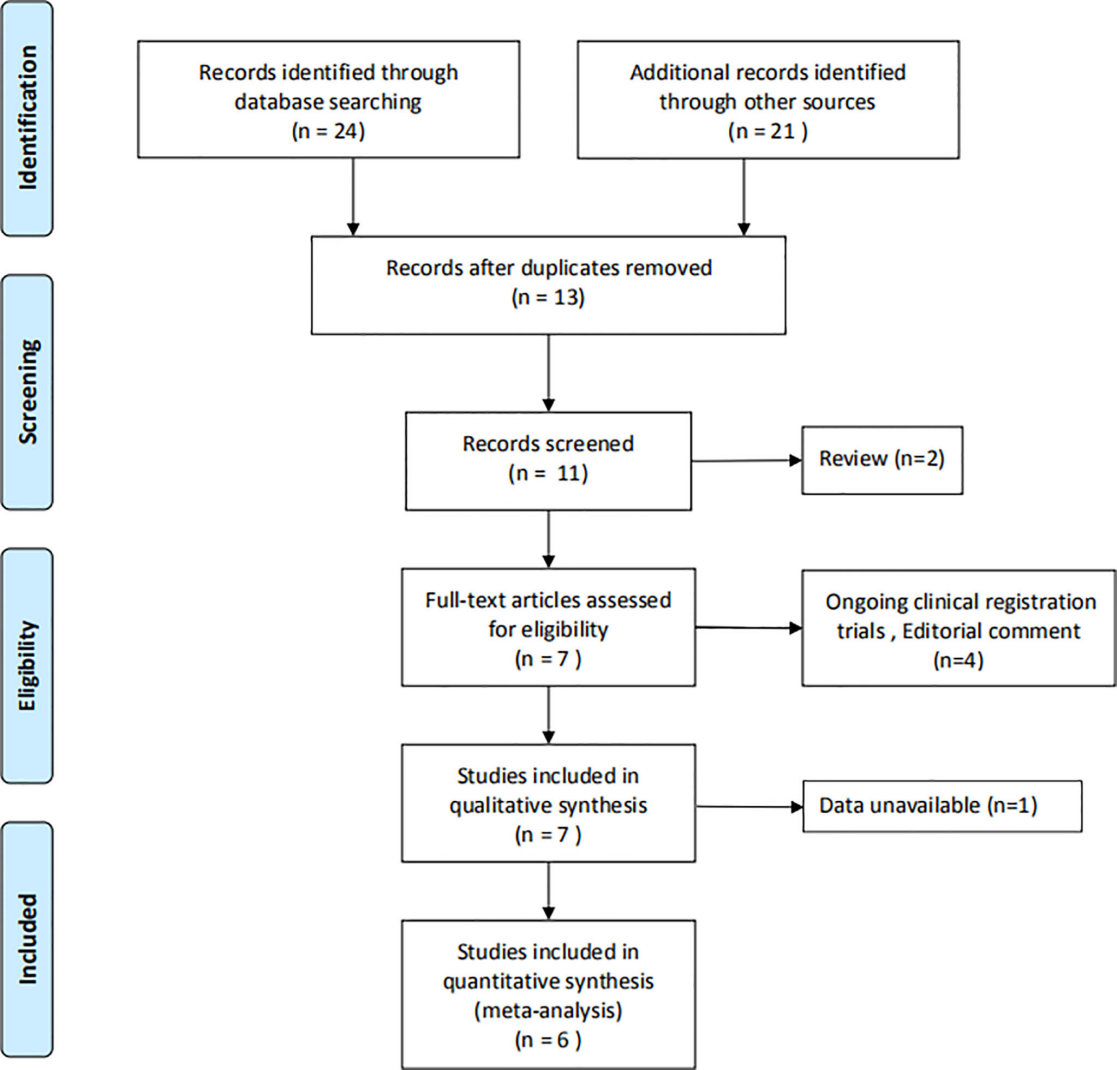
Flowchart of meta-analysis.

Data extraction was performed independently by the two authors using standard data extraction forms. Disagreements were resolved by negotiation with the third reviewer. For each study, the following characteristics were collected: name of the first author, year of publication, follow-up time, anesthetic or pain medications, patient’s information and main outcome indicators. Primary outcome was VAS score including probe insertion, local anesthetic injection, biopsy and 30 min after biopsy. The flow chart showed the filtering of the articles (**Figure 1**).

### Statistical Analysis

The VAS score was compared by mean difference (MD) with 95% confidence intervals (CI). The statistical significance of the summary MD was evaluated by the Z-test. The heterogeneity among the studies was estimated by I^2^-test and chi-square test. If P<0.10, it indicated that significant heterogeneity existed in statistics; and the degree of heterogeneity was classified as follows: I^2^<25%, no heterogeneity; I^2^ 25–50%, moderate heterogeneity; I^2^>50%, large or extreme heterogeneity. When I^2^<50%, the fixed-effects were used to assess the pooled MD; while I^2^>50%, the random-effects were used to assess the pooled MD. This study did not require ethical approval because it was a study using meta-analysis. The quality of included studies was estimated by the risk of bias tool from Cochrane Collaboration for RCTs. For sensitivity analysis, data were extracted separately based on each included study. In order to reduce the influence of confounding factors on the results, subgroup analysis were performed by number of prostate biopsy cores, prostate volume and different biopsy approach.

The software (Review Manager, version 5.3) was used to perform meta-analyses (The Cochrane Information Management System, http://ims.cochrane.org/revman). P<0.05 indicated that significant heterogeneity existed.

## Results

### Eligible Studies

A total of 45 records were obtained by searching six databases. By removing duplicates, reviews, and articles that were not relevant to the question, 11 articles were remained. Then, after full-text screening of these articles, six articles (12, 15–19) were assessed for eligibility. Further evaluations and detailed analysis of the articles were shown in **Figure 1**.

### Literature Analysis

Six articles included 673 prostate cancer cases, with 336 from PPB group and 337 from PNB group. All included studies were RCTs and published in English. The VAS score was directly reported in six studies. Details of all study characteristics are summarized in **Table 1**. According to Cochrane Collaboration, all the included studies are viewed as low risk (**Figures S1** and **S2**).

**Table 1:** The main characteristics of included studies.

First Author, year	Study design	Treatment arms (number of patients)	Anesthetics	Number of prostatic core	Injection Site	Pain Scale
Akpınar et al., 2009 (15)	RCT	PPB(40); PNB(40)	PPB:2 ml of 2% lidocaine PNB:2 mL of 2% lidocaine	12	PPB: pelvic plexus PNB: Base	VAS
Cantiello et al., 2012 (12)	RCT	PPB(90); PNB(90)	PPB: 2.5 ml of a mixture of lidocaine 1% and naropine 0.75%. PNB: 2.5 ml of a mixture of lidocaine 1% and naropine 0.75%.	12	PPB: pelvic plexus PNB: Base	VAS
Ding et al., 2019 (16)	RCT	PPB(81); PNB(83)	PPB: 5 ml 1% lidocaine PNB: 5 ml 1% lidocaine	PPB: 23.1±8.2 PNB: 22.9±8.2	PPB: pelvic plexus PNB: Base	VAS
Jindal et al., 2015 (18)	RCT	PPB(47); PNB(46)	PPB: 2.5 mL 2% lignocaine injection PNB: 2.5 mL 2% lignocaine injection	12	PPB: pelvic plexus PNB: Base	VAS
Kim et al., 2019(1) (17)	RCT	PPB(23); PNB(23)	PPB: 5 ml 2% lidocaine PNB: 5 ml 2% lidocaine	12	PPB: pelvic plexus PNB: Base	VAS
Kim et al., 2019(2) (19)	RCT	PPB(55); PNB(55)	PPB: 3 ml of 2% lidocaine PNB: 3 ml of 2% lidocaine	14	PPB: pelvic plexus PNB: Base	VAS

RCT, randomized controlled trial; PPB, pelvic plexus block; PNB, periprostatic nerve block; VAS, visual analog scale.

### Meta-Analysis VAS Score

#### Probe Insertion

**Figure 2:**
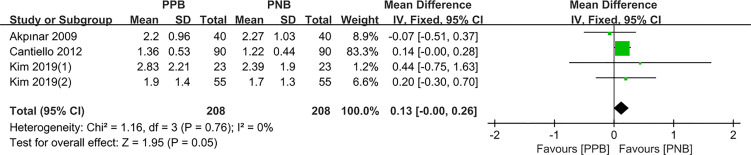
Forest plot comparing VAS score-probe insertion in patients receiving PPB vs PNB.

Four studies compared the pain control of probe insertion between PPB and PNB. Since there was no obvious heterogeneity among these studies (I^2^ = 0%, P = 0.76), fixed-effects model was employed to calculate the pooled MD. The data showed that there was no statistically significant difference of pain control between PPB and PNB (pooled MD = 0.13, 95% CI: 0.00–0.26, Z = 1.95, P = 0.05, **Figure 2**).

#### Local Anesthetic Injection

**Figure 3:**
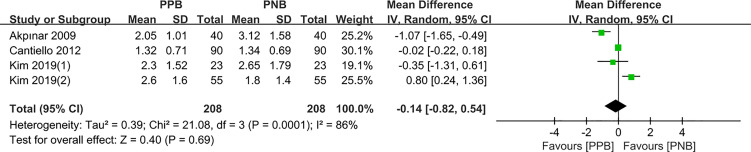
Forest plot comparing VAS score-local anesthetic injection in patients receiving PPB vs PNB.

Four studies enrolling 416 patients reported the Local anesthetic injection VAS score regarding both PPB and PNB. Random-effects model was selected to calculate the combined MD for obvious heterogeneity among the studies (I^2^ =86%, P=0.0001). The results indicated that compared with PNB group, there was no significant difference of pain control in PPB group (pooled MD = −0.14, 95% CI: −0.82 to 0.54, Z = 0.40, P = 0.69, **Figure 3**).

#### During Biopsy

**Figure 4:**
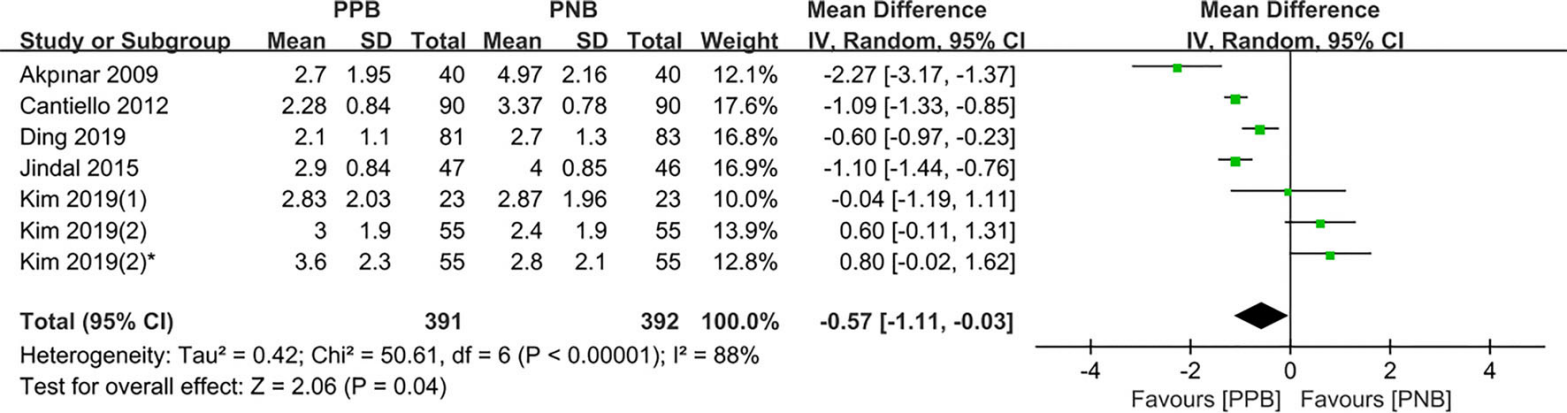
Forest plot comparing VAS score-during biopsy in patients receiving PPB vs PNB.

Six studies compared the pain control of biopsy between PPB and PNB, respectively. Among these studies, Sung Jin Kim et al. (19) compared the VAS score in different biopsy sites. The pooled MD results demonstrated that compared with PNB, PPB was associated with a significant decrease in pain(pooled MD = −0.57, 95% CI: −1.11 to −0.03, Z = 2.06, P = 0.04, **Figure 4**).

#### 30 min after biopsy

**Figure 5:**
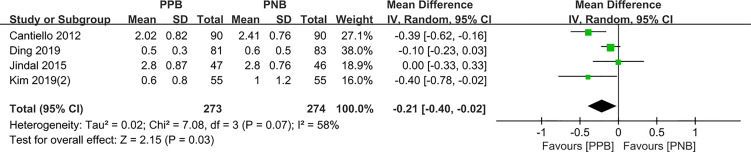
Forest plot comparing VAS score-30 minute after biopsy in patients receiving PPB vs PNB.

Four studies compared the pain control of biopsy between PPB and PNB, respectively. The pooled MD results showed that patients with PPB experienced less pain compared to PNB (pooled MD = −0.21, 95% CI: −0.40 to −0.02, Z=2.15, P = 0.03, **Figure 5**).

#### Subgroup Analysis of Prostate Biopsy

In order to reduce the influence of confounding factors on the results, subgroup analyses were performed by number of prostate biopsy cores, prostate volume and different biopsy approach.

##### Biopsy Cores

Four studies compared the pain control for patients receiving 12-cores biopsy between these two groups. The pooled results showed that PPB was superior to PNB (pooled MD=-1.16, 95% CI: -1.61–0.71, P < 0.00001, **Table 2**). Only one study reported 14-cores and 23-cores prostate biopsy, respectively, and compared with PNB, PPB patients also experienced less pain during biopsy.

**Table 2:** Subgroup analysis of prostate biopsy by number of prostatic core, biopsy approach, or prostate volume.

Subgroup	MD (95% CI)	Heterogeneity, I^2^ (%)	P
Number of prostatic core			
12 cores	−1.16 (−1.61 to −0.71)	69	<0.00001
14 cores	0.60 (−0.11 to 1.31)	NA	0.10
23 cores	−0.60 (−0.97 to −0.23)	NA	0.001
Prostate volume(ml)			
<40	0.42 (−0.18 to 1.03)	0	0.17
40–50	−0.95 (−1.25 to −0.65)	63	<0.00001
>50	−2.27 (−3.17 to −1.37)	NA	<0.00001
Biopsy approach			
Transrectal	−0.81 (−1.45 to −0.18)	87	0.01
Transperineal	−0.60 (−0.97 to −0.23)	NA	0.001

MD, mean difference; NA, not applicable.

##### Prostate Volume

Two studies (17, 19) compared the pain control for patients’ prostate volume less than 40 ml between these two groups. There was no statistical difference for the pain control during biopsy between PPB and PNB. However, three studies (12, 16, 18) and one study (15) reported 40 to 50 ml and more than 50 ml prostate biopsy, respectively, and compared with PNB, PPB patients experienced less pain during biopsy (**Table 2**).

##### Biopsy Approach

Five studies (12, 15, 17–19) and one study (16) reported transrectal and transperineal prostate biopsy, respectively. The pooled results showed that compared with PNB, PPB patients experienced less pain during biopsy, regardless of transrectal or transperineal approach (**Table 2**).

### Meta-analysis-Complications

The reported major complications were urinary retention, hematuria, infection and hemospermia. The pooled results (**Figure S3**) showed that there were no obvious difference in complications between the two groups(urinary retention, OR = 0.76, 95% CI: 0.27–2.14, Z = 0.51, P = 0.61; hematuria, OR = 0.88, 95% CI: 0.55–1.41, Z = 0.53, P = 0.60; infection, OR = 0.75, 95% CI: 0.17–3.39, Z = 0.37, P = 0.71; hemospermia, OR = 1.54, 95% CI: 0.42–5.61, Z = 0.66, P = 0.51).

### Sensitivity analysis

Sensitivity analyses were used to examine the stability of the VAS score results during 12-core biopsy. The results revealed that individual studies cannot influence the final pooled results (**Table 3**). This suggests that the above results were not dominated by any one study.

**Table 3:** Sensitivity analysis of 12-cores prostate biopsy after each study was excluded by turns.

Study omitted	MD (95% CI) for remainders	HeterogeneityI^2^ (%)	P
Cantiello et al., 2012 (12)	−0.83 (−1.08 to −0.58)	65	<0.00001
Jindal et al., 2015 (18)	−0.92 (−1.12 to −0.72)	72	<0.00001
Ding et al., 2019 (16)	−1.06 (−1.26 to −0.87)	36	<0.00001
Kim et al., 2019 (1) (17)	−0.99 (−1.16 to −0.81)	63	<0.00001

## Discussion

Since few RCTs comparing PPB and PNB of ultrasound-guided prostate biopsy were included in the previous systematic review, which may result in unreliable conclusions, so this study uses meta-analysis method to summary all currently published RCTs. The data indicate that compared with PNB, PPB patients have less pain in biopsy and after biopsy, while there was no significant difference in both probe insertion and local anesthetic injection.

Previous meta-analyses (13, 14) only have included two studies, the results showed that PPB significantly reduced pain compared with PNB (MD: −1.09, 95% CI: −1.29 to −0.90, P<0.00001). Our research further confirms the above conclusion, PPB is more effective than PNB at relieving prostate biopsy-related pain. Furthermore, we performed sensitivity analysis, and subgroup analysis according to number of prostate biopsy cores, prostate volume and different biopsy approach, the final results are more complete and reliable.

Nash et al. (20) first reported the use of PNB to diminish pain associated with TRUS-guided prostate biopsy in 1996. However, PNB alone does not completely eliminate the discomfort because it does not affect the pain associated with the TRUS probe. PPB was recently regarded as effective analgesia in prostate biopsy under TRUS guidance (11, 15). Pelvic autonomic plexus is composed of sympathetic and parasympathetic nerve fibers. In fact, the pelvic plexus forms the innervation of the prostate and cavernous nerves that the most caudal part becomes the prostatic plexus (21, 22). The prostatic neurovascular bundle is considered to be the main nerve supply of the prostate (15, 21, 23, 24). Therefore, PPB can produce better anesthetic effect since it blocks the nerve near the prostate and has more nerve fibers. The previous studies reported that PPB under Doppler ultrasound guidance had better analgesic effect than PNB (13, 14). This pooled results are consistent with the above results.

The most common complications were urinary retention, hematuria, infection and hemospermia in prostate biopsy with PPB. However, there were no obvious difference in complications between PNB and PPB. This may be attributed to the precise puncture positioning of Doppler ultrasound reducing damage to the periprostatic vessels (15).

Biopsy is the gold standard for the diagnosis of prostate cancer, and with increasing incidence rate of prostate cancer, more patients with suspected prostate cancer may experience biopsy-related pain. However, using multi-parameter risk assessment model for first prostate biopsy (25), patients with PPB may less pain during biopsy.

In order to get more reliable information than previous studies, we included more patients, and performed subgroup analysis and sensitivity analysis. However, our meta-analysis has several limitations. Firstly, the types, concentrations and doses of anesthetics were not consistent in the included studies, which may influence the effectiveness of pain control. Secondly, different number of prostatic cores may also result in significant heterogeneity, which may have influenced the pooled results. Thirdly, the most of included studies were transrectal-guided prostate biopsy and only one study was transperineal-guided prostate biopsy, so more research is needed to evaluate the pain control with transperineal-guided prostate biopsy in the future. Fourthly, language bias may exist, since all included articles were published in English.

## Conclusion

To sum up, this meta-analysis demonstrates that patients undergoing TUS-guided prostate biopsy under PPB have less pain than that of PNB. At the same time, we should interpret the results with caution because of the heterogeneity among these studies. In future, it also needs more high quality, large samples RCTs to verify.

## Data Availability Statement

The original contributions presented in the study are included in the article/**Supplementary Material**. Further inquiries can be directed to the corresponding author.

## Author Contributions

HD: conceived the idea, carried out the study design, and drafted the manuscript. ZN and HM collected data, performed the data analysis, and drafted the manuscript. All authors contributed to the article and approved the submitted version.

## Funding

The project was funded by the Doctoral research foundation of Lanzhou university second hospital (ynbskyjj2015-1-16) and Cuiying Scientific and Technological Innovation Program of Lanzhou University Second Hospital (PR5108016).

## Conflict of Interest

The authors declare that the research was conducted in the absence of any commercial or financial relationships that could be construed as a potential conflict of interest.

## Supplementary Material

Supplementary Figure 1 Risk of bias graph: each risk of bias item presented as percentages across all included studies.

Supplementary Figure 2 Risk of bias summary: each risk of bias item for each included study.

Supplementary Figure 3 Forest plot comparing complications in patients receiving PPB vs PNB. **(A)**Urinary retention; **(B)** Infection; **(C)** Hematuria; **(D)** Hemospermia.
