# Supplementary figures and images for: Pelvic Plexus Block Versus Periprostatic Nerve Block for Ultrasound-Guided Prostate Biopsy: A Meta-Analysis
Source: Front Oncol. 2021 May 13;11:655906. doi: 10.3389/fonc.2021.655906 (PMC8155474; doi:10.3389/fonc.2021.655906)

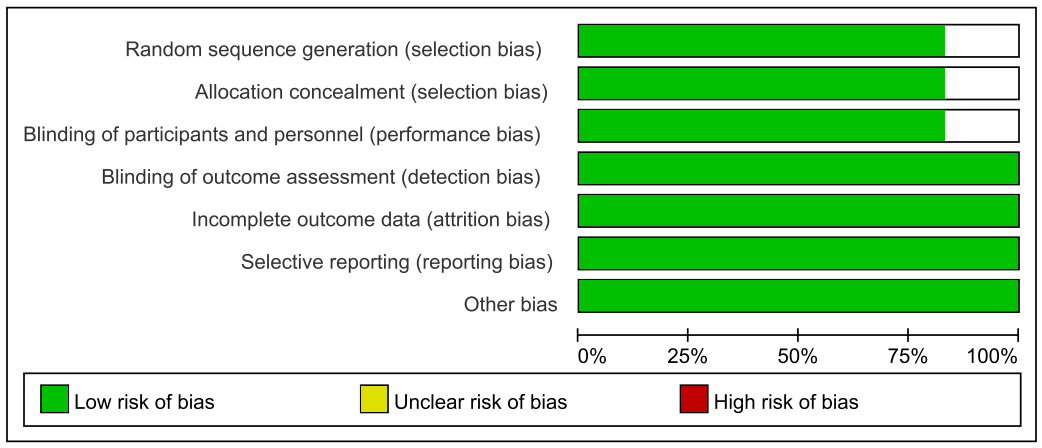

Supplement: Supplementary Figure 1 — Risk of bias graph: each risk of bias item presented as percentages across all included studies. [file Image_1.tif]

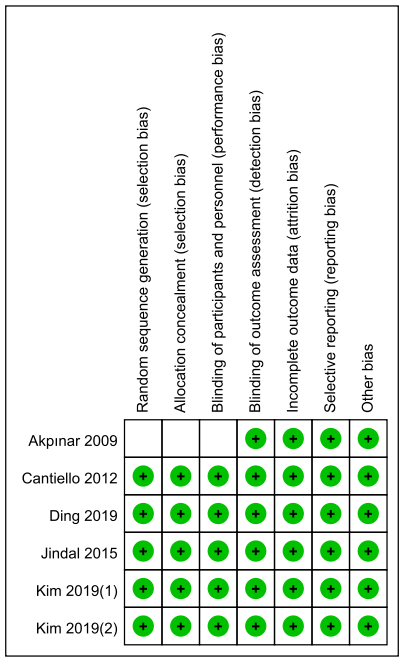

Supplement: Supplementary Figure 2 — Risk of bias summary: each risk of bias item for each included study. [file Image_2.tif]

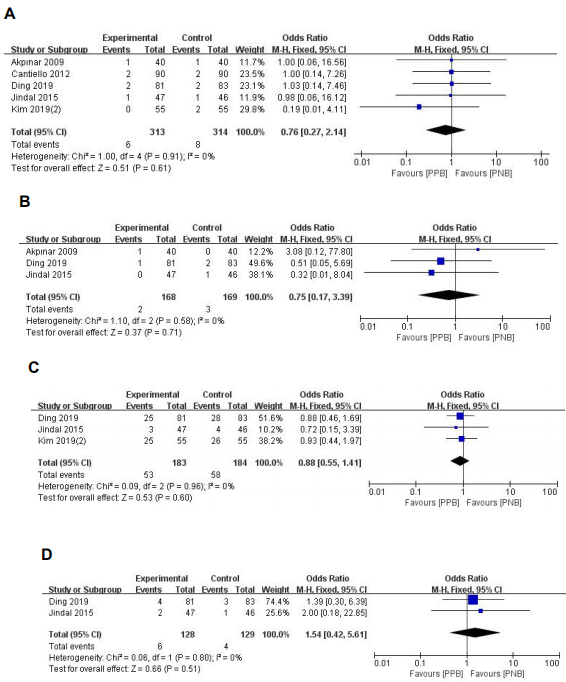

Supplement: Supplementary Figure 3 — Forest plot comparing complications in patients receiving PPB vs PNB. (A)Urinary retention; (B) Infection; (C) Hematuria; (D) Hemospermia. [file Image_3.tif]
